# Viloxazine in the Treatment of Attention Deficit Hyperactivity Disorder

**DOI:** 10.3389/fpsyt.2021.789982

**Published:** 2021-12-17

**Authors:** Amber N. Edinoff, Haseeb A. Akuly, John H. Wagner, Megan A. Boudreaux, Leah A. Kaplan, Shadman Yusuf, Elisa E. Neuchat, Elyse M. Cornett, Andrea G. Boyer, Adam M. Kaye, Alan D. Kaye

**Affiliations:** ^1^Department of Psychiatry and Behavioral Medicine, Louisiana State University Health Science Center Shreveport, Shreveport, LA, United States; ^2^School of Medicine, Louisiana State University Health Shreveport, Shreveport, LA, United States; ^3^School of Medicine, Florida International University, Miami, FL, United States; ^4^Department of Anesthesiology, Louisiana State University Health Shreveport, Shreveport, LA, United States; ^5^Family Psychiatry and Psychology Associates, Cary, NC, United States; ^6^Thomas J. Long School of Pharmacy and Health Sciences, Department of Pharmacy Practice, University of the Pacific, Stockton, CA, United States

**Keywords:** viloxazine, ADHD, non-stimulant, neuropsychiatric disorder, pediatrics, serotonin-norepinephrine modulating agent

## Abstract

Attention deficit hyperactivity disorder (ADHD) is the most common neurodevelopmental disorder in children. Over the past twenty years, research on the disease and its characteristics and treatment options has grown exponentially. The first-line pharmacologic treatment of ADHD is stimulants, which have a response rate of ~70%. With the support of four phase 3 studies involving more than 1,000 pediatric patients 6–17 years old, the FDA has approved the non-stimulant, serotonin-norepinephrine modulating agent (SNMA) viloxazine in an extended-release capsule (viloxazine ER) for treatment of ADHD in children aged 6–17. Viloxazine modulates serotonergic activity as a selective 5-HT2_2B_ receptor antagonist and 5-HT_2C_ receptor agonist and moderately inhibits norepinephrine transporter (NET), thus blocking the reuptake of norepinephrine. A phase 2 study by Johnson et al. found that once-daily dosing of viloxazine ER in 200, 300, or 400 mg dosages in children with ADHD for eight weeks resulted in a statistically significant reduction of ADHD-RS-IV total score. A *post hoc* analysis of data from four phase 3, randomized, placebo-controlled, double-blind, three-arm, clinical trials by Faraone et al. found that early response to viloxazine treatment, defined as a change in ADHD-RS-5 total score at week 2, best predicted the treatment response at week 6 [75% positive predictive power (PPP), 75% sensitivity]. Proper treatment of the symptoms and comorbidities associated with ADHD is crucial in improving a patient's quality of life, cognitive function, and overall therapeutic outcomes. Viloxazine's mechanism of action, clinical effects, and limited side effect profile point toward the drug's relevance in the treatment of ADHD.

## Introduction

According to DSM-5, attention deficit hyperactivity disorder (ADHD) is a “chronic, neurologically-based illness characterized by a persistent pattern of inattention and/or hyperactivity and impulsivity that are more inappropriate or disruptive than those in other children of a comparable age resulting in functional impairment in multiple settings, and these behaviors have been present for at least six months (1).” The core symptoms of ADHD are inattentiveness, impulsivity, and/or motor unrest, which are symptoms also prevalent in other neuropsychological disorders, such as conduct disorder and depression (2).

ADHD is the most common neurodevelopmental disorder in children (3). Over the past twenty years, research on the disease and its characteristics and treatment options has grown exponentially (2). Though ADHD has not become any more prevalent in the general population in recent years, the disorder has become increasingly well-recognized, likely due to increased recognition of the impact of ADHD on functioning, advances in diagnostic methodology and technology, and interest from pharmaceutical companies (2).

In 2013, the DSM-5 revised the criteria for diagnosis, including raising the age limit for onset of qualifying symptoms from before six years of age to before 12 years of age, requiring the presence of symptoms in at least two settings, and requiring five rather than six symptoms for diagnosis in patients age 17 and older (2). Despite the previous misconception, ADHD often persists into adulthood, along with new comorbidities, consequences, and treatment options (1).

The first-line pharmacologic treatment of ADHD is stimulants, which have a response rate of ~70% (2). Amphetamine-based medications such as Adderall and Ritalin are powerful sympathomimetic drugs and can after mood, appetite, growth, blood pressure, pulse, and sleep patterns. Aggressiveness, hallucinations, and increased anxiety can be associated with the use of these medications. This is why the use of non-stimulant medications may be a good alternative for some patients. Any medication should be combined with behavioral management for best effect (2). With the support of four phase 3 studies involving more than 1,000 pediatric patients 6–17 years old, the FDA has approved the non-stimulant, serotonin-norepinephrine modulating agent (SNMA) viloxazine in an extended-release capsule (viloxazine ER) for treatment of ADHD in children aged 6–17 (4). This manuscript will look at this medication but first will look at ADHD along with its current treatment.

### ADHD Presentation

ADHD characteristically presents in childhood with persistently high levels of hyperactivity, impulsivity, and/or inattention. ADHD places students at a particular disadvantage with academic performance. They are often unable to process information as quickly as their peers and are more likely to perform poorly on standardized exams, likely related to inattentiveness (5). The impulsivity associated with ADHD increases the risk for unsafe behaviors, such as spontaneous sexual behavior and motor vehicle collisions (6, 7). ADHD also often presents with other comorbid psychiatric disorders, such as major depressive disorder (MDD) and anxiety disorders (8). Examples of clinically significant inattentiveness include overlooking details and turning in work riddled with careless mistakes; difficulty maintaining focus during tasks, resulting in incomplete assignments and missed deadlines; and frequently misplacing items like keys, wallets, or cellphones. Examples of hyperactivity and impulsivity in ADHD include being “fidgety” and having problems sitting still for prolonged periods of time. Individuals may talk excessively or inappropriately, blurt out answers, and interrupt conversations (9).

### Pathophysiology

ADHD is primarily related to the imbalance of neurotransmitters, especially dopamine (DA) and norepinephrine (NE), resulting in inattention and hyperactivity. One leading theory specifies hypoactivity of NE and DA in the prefrontal cortex (PFC), which is involved in attention, formulating plans, organizing thoughts, and suppressing the processing of irrelevant and distracting stimuli, allowing a person to remain focused on a specific task (10). Magnetic resonance imaging (MRI) studies have shown decreased frontal lobe volume in individuals with ADHD, and functional MRI (fMRI) studies have demonstrated hypoactivity (11, 12).

The caudate nucleus, corpus callosum, and cerebellar vermis may also be decreased in volume in individuals with ADHD (13). DA is strongly implicated in the neural circuits involved with behavior reinforcement and reward processing in the basal forebrain, so that DA hypoactivity may contribute to impulsivity (19, 20).

## Current Treatment of ADHD

ADHD is treated with pharmacological and non-pharmacological interventions, including psychotherapeutic and dietary approaches. The two classes of psychostimulants, methylphenidate, and amphetamine, are the first-line pharmacological treatments for ADHD (14). Second-line pharmacological treatments include atomoxetine, guanfacine, and clonidine; however, these medications are less effective than psychostimulants and thus less frequently used (15). Methylphenidate is structurally similar to NE and DA. Thus, it acts by binding to the monoamine transporters NET and DAT, inhibiting NE and DA reuptake and increasing the availability of these neurotransmitters in the synapse (14).

Amphetamines demonstrate three main mechanisms of action. First, they also bind to monoamine transporters, norepinephrine transporter (NET), and dopamine transporter (DAT) to prevent the reuptake of their respective neurotransmitters. Second, amphetamines allow trace-amine associated receptor 1 (TAAR1) to phosphorylate the DAT transporter, allowing for ceased transport or inversing the efflux of dopamine. Third, amphetamines may enter presynaptic monoamine vesicles, causing an efflux of neurotransmitters toward the synapse (14).

Atomoxetine (otherwise known as Strattera®) is a non-stimulant that acts as a norepinephrine reuptake inhibitor, increasing the amount of norepinephrine available in the synapse (16). Atomoxetine is commonly prescribed when stimulants are contraindicated, such as in individuals with substance abuse disorders, bipolar disorder, or Tourette syndrome who may be at higher risk of mood destabilization (14). Antidepressants have also been used to treat ADHD to increase serotonin, dopamine, and/or norepinephrine by blocking the reuptake (16).

In terms of non-pharmacologic interventions, therapy and cognitive training techniques also show promise in the treatment of ADHD symptoms.

Behavioral and cognitive-behavioral therapies alter the child's behavior through social learning principles (17). Cognitive training involves strengthening the patient's working memory and attention via schedules, social skills training, verbal self-instruction, and problem-solving strategies.

### Viloxazine

With the support of four phase 3 trials of more than 1,000 pediatric patients, the FDA has approved the SNMA viloxazine in an extended-release (ER) capsule for the treatment of ADHD in children aged 6–17 (4, 18). Though not considered a stimulant medication, viloxazine produces amphetamine-like CNS stimulant effects without evidence of drug dependence (18). Usually prescribed for depression, viloxazine has similar therapeutic efficacy to imipramine and amitriptyline, without the sedative anticholinergic or adrenergic effects typical of tricyclic antidepressants (18). As with other antidepressants, higher rates of suicidal thoughts and behaviors were reported in pediatric patients treated with the drug than those treated with placebo (19). The most common adverse reactions are somnolence, decreased appetite, fatigue, nausea, vomiting, insomnia, and irritability (20). Other warnings and precautions include possible activation of mania or hypomania in patients with bipolar disorder. Patients should use caution when operating hazardous machinery or driving a car due to potential somnolence and fatigue (20).

### Mechanism of Action

Viloxazine is an SNMA, such that its mechanism of action (MoA) is unique compared to those of other ADHD and depression pharmacotherapies (21). Viloxazine modulates serotonergic activity as a selective 5-HT2_2B_ receptor antagonist and 5-HT_2C_ receptor agonist and moderately inhibits NET, thus blocking the reuptake of norepinephrine (20). The 5-HT receptors, in general, have a variety of functions such as the modulation of GABA, glutamate, dopamine, epinephrine/epinephrine, and acetylcholine. It also works on many hormones including oxytocin, prolactin, vasopressin, cortisol, corticotropin, and substance P. In the CNS, %-HT receptors exert influence on almost every major function including memory, cognition, emotion, perception, mood, and even consciousness (22). The 5-HT2 family in general have been targets of many psychotropic medications, such as antidepressants, antipsychotic, anorectic, anti-insomnia. And anxiolytic medications (22).

The only moderate inhibitory activity of viloxazine at NET is consistent with the observed low rate of cardiac effects, more typical of primary norepinephrine reuptake inhibitors (NRIs) (21). Viloxazine's modulation of 5-HT and NE supports its therapeutic relevance in treating neuropsychiatric disorders impacted by monoaminergic transmission, such as ADHD and depression (21). NE and DA dysregulation contribute to inattention and deficits in executive functioning in ADHD. Recently, 5-HT has been recognized as a potential additional therapeutic target in ADHD, supported by rodent models, further pointing toward viloxazine's relevance in treating ADHD (21).

### Dosing, Pharmacokinetics, and Pharmacodynamics

Viloxazine ER is available in 100, 150, and 200 mg capsules. In pediatric patients aged 6–11 years of age, the recommended starting dose is 100 mg orally once daily and can be titrated in increments of 100 mg weekly to the maximum recommended dosage of 400 mg once daily, depending upon patient response and medication tolerability (20). In pediatric patients aged 12–17, the recommended starting dose is 200 mg orally once daily for the first week of administration and can be titrated to the maximum daily dose of 400 mg (20).

The bioavailability of viloxazine is estimated to be ~88%, and the time to peak plasma concentration is 5 h for one 200 mg dose. The drug is highly protein-bound (76–82%), and the mean half-life of the viloxazine ER is 7.02 ± 4.74 h. Viloxazine undergoes metabolism via the cytochrome-P450 enzyme CYP2D6 and the UDP-glucuronosyltransferase enzymes UGT1A9 and UGT2B15. The inhibitory constant (K_i_) for viloxazine at the norepinephrine transporter (NET) is 0.63 μM, while the half-maximal inhibitory concentration (IC50) is 0.2 μM (23).

Patients taking drugs metabolized by the CYP1A2 enzyme may require dosing reductions, as viloxazine is a potent CYP1A2 inhibitor. As with other serotonergic antidepressants, concomitant use of viloxazine within fourteen days of the discontinuation of a monoamine oxidase inhibitor (MAOI) is contraindicated, as administration of these drugs together could precipitate a hypertensive crisis. Viloxazine is primarily excreted renally as the major plasma metabolite 5-hydroxy-viloxazine glucuronide, such that special dosing and titration are crucial in patients with renal impairment (18, 20). Viloxazine is contraindicated in patients with severe hepatic insufficiency due to its pharmacokinetic profile (23). Coadministration of viloxazine ER and methylphenidate, a stimulant used in treating ADHD, does not affect the pharmacokinetic profile of either drug, and the combination may be well-tolerated in appropriate patient populations (24).

### Clinical Studies

A study by Nasser et al. on the effect of viloxazine ER on the QTc interval of healthy adults (25). This study was a phase I, 14-day, randomized, double-blind (except for moxifloxacin control), 3-treatment, 3-period, 6-sequence, crossover design study in healthy adults examining the ECG effects of viloxazine ER. The authors examined ECG and pharmacokinetic measurements, including the effect of a supratherapeutic dose of viloxazine ER on cardiac repolarization (OTc) in healthy adults. There was a statistically significant negative slope (*P* = 0.0012) between the Δ QtcI and viloxazine Cp and between 5-OH-VLX-gluc Cp and ΔQTcI (*P* = 0.0007), and secondary time point analyses did not show an effect of viloxazine ER on QtcI. The authors found that viloxazine ER does not have an impact on cardiac repolarization and/or another electrocardiogram (ECG) parameters, such that it does not increase the risk for cardiac arrhythmias.

A phase 3 study by Nasser et al. measured the change in the ADHD-Rating Scale-5 (ADHD-RS-5) total scores in schoolchildren with ADHD prescribed viloxazine ER (26). This was a phase 3, 6-week, randomized, double-blind, placebo-controlled trial, designed to examine the efficacy and safety of viloxazine ER for the treatment of ADHD of school-age children. The authors examined the primary efficacy endpoint based on change from baseline (CFB) at end of study (EOS) in the ADHD-RS-5 total score. 477 subjects with similar demographic and baseline characteristics were randomized to treatment with viloxazine ER 100 or 200 mg daily or placebo. There was a statistically significant improvement in the ADHD-RS-5 total score in both the viloxazine ER 100-and 200-mg/day treatment groups compared to placebo (*P* = 0.0004 and *P* = 0.0244, respectively). There were also improvements in the CGI-I scores (*P* = 0.0020 and *P* < 0.0001), Conners 3-PS composite T-score (*P* = 0.0003 and *P* = 0.0002), and WIFIRS-P Total average score (*P* = 0.0019 and *P* = 0.0002) compared to placebo. There were also improvements in the CGI-I scores (*P* = 0.0020 and *P* < 0.0001), Conners 3-PS composite T-score (*P* = 0.0003 and *P* = 0.0002), and WIFIRS-P Total average score (*P* = 0.0019 and *P* = 0.0002) when compared to placebo. The authors found that the drug significantly improved ADHD symptoms. Adverse events related to treatment were mild and included headache, somnolence, and decreased appetite.

A phase 2 study by Johnson et al. found that once-daily dosing of viloxazine ER in 200, 300, or 400 mg dosages in children with ADHD for eight weeks. This study was a 8-week, randomized, double-blind, placebo-controlled trial, designed to examine the efficacy and safety of viloxazine ER in children with ADHD. The authors examined ADHD-RS scores, CGI-S and CGI-I scores, and safety assessments, including lab and ECG measurements, suicidality monitoring, and AEs. 222 participants aged 6–12 years old with ADHD were randomized to placebo or one of the following viloxazine ER dosage groups: 100, 200, 300, or 400 mg/day. The average ADHD-RS-IV total score decreased in all the dosing and placebo groups. In the viloxazine ER 200, 300, and 400 mg dosage groups, the least square (LS) mean CFB in ADHD-RS-IV was significant (*p* = 0.031, 0.027, and 0.021, respectively) compared to the placebo (27). The most frequent AEs were somnolence, headache, and decreased appetite, and the TEAE incidence rate was similar for all treatment groups. There were no significant trends in vital signs, ECG results, or clinical lab tests.

A single-center, crossover, open-label trial conducted by Faison et al. found that the coadministration of viloxazine ER and methylphenidate. This was a single-center, crossover, open-label trial in healthy adults, designed to examine the pharmacokinetics of co-administered viloxazine ER and methylphenidate. The authors examined the pharmacokinetics of 700 mg viloxazine ER alone, 36 mg methylphenidate alone, and combination viloxazine ER (700 mg) + methylphenidate (36 mg). Active drug amount was measured using chromatographic tandem mass spectrometry. Safety assessments such as vitals, ECGs, AEs, and clinical lab evaluations were assessed. For viloxazine ER alone, the following results were obtained: *C*_max_ 100.98%, AUC_*t*_ 98.62%, and AUC_∞_ 98.96%. For methylphenidate alone, the following results were obtained: *C*_max_ 103.55%, AUC_*t*_ 106.67%, and AUC_∞_ 106.61%. The AEs were relatively mild, did not cause any patient to discontinue the study, and included dizziness, nausea, somnolence, and insomnia. Changes in clinical lab tests, vital signs, physical exam, and suicidality were clinically insignificant. The pharmacokinetics did not impact the pharmacokinetics of either drug. The viloxazine ER and methylphenidate combination for ADHD appeared to be safe for administration with very mild side effects, such as dizziness, nausea, somnolence, and insomnia (24).

A *post hoc* analysis of data from four phase 3, randomized, placebo-controlled, double-blind, three-arm clinical trials by Faraone et al. was performed. This *hoc* analysis of data from four phase 3, randomized, placebo-controlled, double-blind, 3-arm clinical trials examining how early response to viloxazine ER predicts efficacy in pediatric patients. Treatment response was defined as having a ≥50% reduction in ADHD-RS-5 total score at Week 6. The CGI-score at weeks 1–3 and target dose were also assessed. The change in ADHD-RS-5 total score at week 2 best predicted the treatment response at week 6 (75% PPP, 75% sensitivity). Of the study sample of 774 treated subjects, 342 subjects (44%) were categorized as responders to the treatment. A patient's ADHD-RS-5 scores at week 2 can be used to make clinically useful predictions of response to viloxazine. The week 2 data show higher levels of PPP that do not decrease excessively as sensitivity increases (28).

A *post hoc* analysis by Nasser et al. data from four trials correlated ADHD-RS-5 and Weiss Functional Impairment Rating Scale-Parent (WFIRS-P) scores with CGI scores. CGI scales are more intuitive assessments that can more easily assess the clinical implications of treatment. For the “improved” and “very much improved” ratings on the CGI-I, there were score improvements on the ADHD-RS-5 of ~55 and 80%, respectively, and improvements on the WFIRS-P of ~40 and 70%, respectively (29).

Another study by Nasser et al. utilized ADHD-RS-5 total score, CGI, and assessments of adverse events in adolescents with ADHD prescribed viloxazine ER. This was a phase 3, randomized, double-blind, placebo-controlled trial, designed to examine the efficacy and safety of viloxazine ER 200 or 400 mg vs. placebo in adolescents aged 12–17 with ADHD. There was a statistically significant improvement in the ADHD-RS-5 total score in the viloxazine ER 200 and 400 mg groups (*p* = 0.0232 and *p* = 0.0091, respectively) compared to the placebo. There was also a statistically significant improvement in CGI-score for both the 200 and 400 mg dose groups (*p* = 0.0042 and *p* = 0.003, respectively) compared to placebo. There were no significant changes found in the Conners 3-PS Composite T-score (*p* = 0.6854, *p* = 0.0518, respectively) or WFIRS-P total average score (*p* = 0.2062, *p* = 0.0519, respectively). The most common AEs included decreased appetite, fatigue, headache, somnolence, and nausea. The authors concluded that the drug to be well-tolerated and to significantly improve ADHD symptoms (30). See Table 1 for a summary of the above studies.

**Table 1:** Clinical safety and efficacy.

**References**	**Groups studied and intervention**	**Results and findings**	**Conclusions**
Nasser et al. (25)	Phase I, 14-day, randomized, double-blind (except for moxifloxacin control), 3-treatment, 3-period, 6-sequence, crossover design study in healthy adults examining the ECG effects of viloxazine ER.	Viloxazine ER does not have an impact on cardiac repolarization and/or another electrocardiogram (ECG) parameters, such that it does not increase the risk for cardiac arrhythmias	Viloxazine ER was found to have no effect on cardiac repolarization or other ECG parameters in healthy adults, suggesting it does not increase the risk for cardiac arrhythmias.
Nasser et al. (26)	A phase 3 study measured the change in the ADHD-Rating Scale-5 (ADHD-RS-5) total scores in schoolchildren with ADHD prescribed viloxazine ER	Randomized to treatment with viloxazine ER 100 or 200 mg daily or placebo. There was a statistically significant improvement in the ADHD-RS-5 total score in both the viloxazine ER groups compared to placebo (*P* = 0.0004 and *P* = 0.0244, respectively).	Significantly improved ADHD symptoms. Adverse events related to treatment were mild and included headache, somnolence, and decreased appetite
Johnson et al. (27)	Phase 2, 8-week, randomized, double-blind, placebo-controlled trial, designed to examine the efficacy and safety of viloxazine ER in children with ADHD.	Statistically significant reduction of ADHD-RS-IV total score and improvement in Clinical Global Impression – Severity and Improvement (CGI-S and CGI-I) scores, thus improving ADHD symptoms.	The results were dosage-dependent, as the children in the 400-mg/day dose had the greatest reduction of ADHD symptoms. Treatment was well-tolerated.
Faison et al. (24)	Single-center, crossover, open-label trial in healthy adults, designed to examine the pharmacokinetics of co-administered viloxazine ER and methylphenidate.	At each timepoint, the combination of viloxazine and methylphenidate did not alter the relative bioavailability compared to the drugs being administered alone. The AEs were relatively mild, did not cause any patient to discontinue the study, and included dizziness, nausea, somnolence, and insomnia. Changes in clinical lab tests, vital signs, physical exam, and suicidality were clinically insignificant.	It was found that the combination of viloxazine ER (700 mg) + methylphenidate (36 mg) was safe and well-tolerated. The coadministration of the drugs did not alter the pharmacokinetics compared to those of each drug alone.
Faraone et al. (28)	Machine learning *post hoc* analysis of data from four phase 3, randomized, placebo-controlled, double-blind, 3-arm clinical trials Treatment response was defined as having a ≥50% reduction in ADHD-RS-5 total score at Week 6.	The change in ADHD-RS-5 total score at week 2 best predicted the treatment response at week 6 (75% PPP, 75% sensitivity).	A patient's ADHD-RS-5 scores at week 2 can be used to make clinically useful predictions of response to viloxazine. The week 2 data show higher levels of PPP that do not decrease excessively as sensitivity increases.
Nasser et al. (29)	*post-hoc* analyses of data from four phase 3 trials in children/adolescents with ADHD who were treated with viloxazine ER.	In participants who showed improvement, a one-level CGI-I change was correlated with 0.2–0.5-point and 10–15 point changes on the WFIRS-P and ADHD-RS-5 scales, respectively. For the “improved” and “very much improved” ratings on the CGI-I, there were score improvements on the ADHD-RS-5 of ~55 and 80%, respectively, and improvements on the WFIRS-P of ~40 and 70%, respectively.	Improvement on ADHD symptom scales following treatment with viloxazine ER translated into clinically significant improvement for many patients. These *post-hoc* analyses helped create clinical benchmarks to interpret the results of ADHD-Rs-5 and WFIRS-P via their correlation with CGI scores in individuals with ADHD.
Nasser et al. (30)	Phase 3, randomized, double-blind, placebo-controlled trial, designed to examine the efficacy and safety of viloxazine ER 200 or 400 mg vs. placebo in adolescents aged 12–17 with ADHD.	There was a statistically significant improvement in the ADHD-RS-5 total score in the viloxazine ER (200 and 400 mg groups) compared to the placebo. The most common AEs included decreased appetite, fatigue, headache, somnolence, and nausea.	Viloxazine ER 200 and 400 mg doses were efficacious and safe in adolescents with ADHD.

## Conclusion

ADHD, the most common neurodevelopmental disorder in children, characteristically presents in childhood with persistently high levels of hyperactivity, impulsivity, and inattention (3, 9). ADHD is largely due to the imbalance of neurotransmitters, especially DA and NE in the PFC, leading to inattention and hyperactivity (10). Proper treatment of the symptoms and comorbidities associated with ADHD is crucial in improving a patient's quality of life, cognitive function, and overall therapeutic outcomes (1). ADHD is treated with pharmacological and non-pharmacological interventions, including psychotherapeutic and dietary approaches (11).

Over the past twenty years, research on ADHD and its characteristics and treatment options has grown exponentially, resulting in new pharmacologic options, including viloxazine (2). Viloxazine, an SNMA, is a recently FDA-approved non-stimulant pharmacological option for children ages 6–17 for the treatment of ADHD (5). Viloxazine acts as a selective 5-H2_2B_ receptor antagonist and 5-HT_2C_ receptor agonist and moderately inhibits norepinephrine transporter (NET), thus blocking the reuptake of norepinephrine (20). Viloxazine's mechanism of action, clinical effects, and limited side effect profile point toward the drug's relevance in the treatment of ADHD (21). This drug could be especially useful in those who have co-morbid anxiety disorders with ADHD and cannot tolerate the anxiety-increasing effects of stimulants, which have been the mainstay of treatment. This is a promising step forward for the treatment of ADHD in both children and adults and can change the quality of life in those with the disorder.

## Author Contributions

AE, MB, LK, and SY are responsible for the writing of the manuscript. HA, JW, EN, EC, AG, ADK, and AMK are responsible for editing. AE was also responsible for the idea concept and design of manuscript. All authors contributed to the article and approved the submitted version.

## Conflict of Interest

The authors declare that the research was conducted in the absence of any commercial or financial relationships that could be construed as a potential conflict of interest.

## Publisher's Note

All claims expressed in this article are solely those of the authors and do not necessarily represent those of their affiliated organizations, or those of the publisher, the editors and the reviewers. Any product that may be evaluated in this article, or claim that may be made by its manufacturer, is not guaranteed or endorsed by the publisher.
